# Probiotic supplementation attenuated early-life chemotherapy-induced brain development impairment in mice

**DOI:** 10.3389/fnbeh.2025.1697727

**Published:** 2025-12-17

**Authors:** Aihua Tan, Jie Chen, Juan Zhang, Jianbin Tong, Zhibin Jiang

**Affiliations:** 1 Department of Anesthesiology, The Third Xiangya Hospital of Central South University, Changsha, Hunan, China; 2 Hunan Province Key Laboratory of Brain Homeostasis, Third Xiangya Hospital, Central South University, Changsha, Hunan, China; 3 Department of Breast Surgery, Tangshan People’s Hospital, Tangshan, Hebei, China; 4 The Third Xiangya Hospital of Central South University, Changsha, Hunan, China

**Keywords:** early life chemotherapy, cognitive impairment, probiotics, acute lymphoblastic leukemia, anxiety

## Abstract

**Background:**

Brain dysfunction is a common post-chemotherapy sequela in acute lymphoblastic leukemia (ALL) survivors and is associated with poor academic performance and reduced work ability. The prevention of brain dysfunction in ALL survivors remains a clinical challenge. In this study, we evaluated the preventive effects of probiotics on chemotherapy-induced brain development damage in a preclinical setting.

**Methods:**

The clinical ALL chemotherapy setting was mimicked by intraperitoneally injecting doxorubicin into 4-week-old mice once every 3 days for 2 weeks. Probiotics were administered in the drinking water from the beginning of chemotherapy until adulthood. Behaviors at adulthood were assessed using open field, elevated plus maze, novel object recognition, and Barnes maze tests. Fecal microbiota composition was analyzed using 16S ribosomal RNA (rRNA) gene sequence. Hippocampal neurogenesis was assessed using EdU staining and DCX immunostaining. Synaptic protein expressions were detected using Western blotting.

**Results:**

Early-life chemotherapy induced cognitive dysfunction in adulthood, as demonstrated by impairments in the novel object recognition and Barnes maze tests, but it did not significantly alter anxiety-like behavior in the elevated plus maze. Early-life chemotherapy also induced fecal microbiota dysbiosis both at the end of chemotherapy and in adulthood. Probiotic supplementation alleviated early-life chemotherapy-induced cognitive dysfunction and fecal microbiota dysbiosis in adulthood. In addition, probiotic supplementation also alleviated early-life chemotherapy-induced hippocampal neurogenesis impairments and synaptic protein loss.

**Conclusion:**

Probiotic supplementation can improve early-life chemotherapy-induced brain development impairments in mice by modulating hippocampal neurogenesis.

## 1 Introduction

Acute lymphoblastic leukemia (ALL) is the most common childhood cancer, accounting for nearly 28–30% of all cases (Siegel et al., 2020; Amitay and Keinan-Boker, 2015). With advances in chemotherapy, the 5-year overall survival rate for childhood ALL has increased to 90% (Dixon et al., 2020); the cumulative incidence of all-cause late mortality at 20 years after diagnosis is only 6.6% (Dixon et al., 2020). Among ALL survivors, post-ALL sequelae, including brain dysfunction and cardiovascular disease, have emerged as major clinical challenges (Lipshultz et al., 2013; George et al., 2021; Onzi et al., 2022; Liu et al., 2023).

Brain dysfunction is a common post-ALL sequela and a highly prevalent complication of chemotherapy. Approximately 17–54% of childhood ALL survivors experience difficulties in executive function, including impairments in working memory and cognitive flexibility (van der Plas et al., 2021a). Up to 62% of survivors show attention deficits (Chiou et al., 2019), and 15–43% exhibit reduced processing speed (Liu et al., 2018). Consequently, brain dysfunction negatively affects ALL survivors. To date, non-pharmacological interventions such as psychological therapies (Duval et al., 2022; Zeng et al., 2024), behavioral training (Cherrier et al., 2013; Janelsins et al., 2016), and cognitive training (Hardy et al., 2013; Conklin et al., 2015) have been widely used to treat brain dysfunction in ALL survivors. However, poor compliance and limited effectiveness of these inventions remain major challenges (Wefel et al., 2015). Therefore, new methods are needed.

The microbiota plays an important role in brain development and in maintaining normal adult brain function (Cryan et al., 2019; Margolis et al., 2021; Obata and Pachnis, 2016). Our previous study showed that probiotic supplementation prevents the occurrence of chemotherapy-related cognitive impairment in patients with breast cancer by modulating plasma metabolites, including p-Mentha-1,8-dien-7-ol (Juan et al., 2022). Microbial reconstitution can reverse cognitive impairment and synaptic defects in rat offspring induced by pregnancy stress (Chen et al., 2024). As we know, the developing brain is different from the adult brain. It is still unclear whether probiotic supplementation can attenuate early-life chemotherapy-induced brain development impairment in ALL survivors.

Pegylated liposomal doxorubicin is commonly used in chemotherapy of ALL (Malard and Mohty, 2020). In this study, we injected pegylated liposomal doxorubicin into mice during early life to mimic childhood chemotherapy for ALL, and we evaluated the preventive effects of probiotics on brain damage and the mechanisms involved.

## 2 Materials and methods

### 2.1 Animals

The experimental protocols were carried out in accordance with the guidelines for the use of laboratory animals at Central South University and received approval from the Animal Ethics Committee of Central South University (Approval Number: CSU-2024-0202). C57BL/6 male mice (4-week-old) were purchased from Central South University. All animals were housed in pathogen-free cages with free access to food and water. Suitable temperature and humidity levels and regular light cycles were guaranteed.

### 2.2 Grouping and processing

The 4-week-old C57BL/6 male mice were randomly divided into three groups: control group, chemotherapy group, and probiotic group. The mice in the chemotherapy and probiotic groups were intraperitoneally injected with 2 mg/kg doxorubicin (DOX) once every 3 days for 2 weeks. The choice of the DOX dose was based on previous studies (Zhao et al., 2023). Mice in the probiotic group were fed daily with probiotics, and probiotic capsules (BIFICO, Xinyi Pharmaceutical, Shanghai, China) containing *Bifidobacterium longum* (1.0 × 10^7^ CFU/210 mg), *Lactobacillus acidophilus* (1.0 × 10^7^ CFU/210 mg), and *Enterococcus faecalis* (1.0 × 10^7^ CFU/210 mg) were added to the drinking water at a dosage of one capsule per mouse per day for 4 weeks.

### 2.3 Behavioral tests

#### 2.3.1 Open field

The open field test is used to detect the exploration level and the autonomous activity ability of animals. All animals were placed in the testing room 30 min before the start of the experiment so that they could familiarize themselves with the environment. When the test began, the animals were placed in an opaque cube box and allowed to explore freely for 10 min. The time spent exploring the central area was recorded.

#### 2.3.2 Elevated plus maze

The elevated plus maze is used to assess anxiety-like behavior in animals. The experimental setup had a pair of open arms and a pair of closed arms, and each animal was placed in the area where the open and closed arms met, allowing it to explore freely for 5 min. The time spent by the animals in the open and closed arms was recorded separately.

#### 2.3.3 Novel object recognition test

The novel object recognition test was used to assess memory and cognitive abilities in the animals. As previously reported (Chen et al., 2024), the experiment was divided into two phases: training and testing. During the training phase, two identical objects were placed equidistant from the center of the area in an empty box, and the animals were allowed to explore freely for 10 min. During the testing phase, one of the familiar objects in the empty box was replaced with a new object of a different shape, and the animals were allowed to explore freely for 10 min. The time spent by the animals exploring the new and the old objects during the test phase was recorded separately to calculate the recognition index using the formula: new object exploration time/(new object exploration time + old object exploration time).

#### 2.3.4 Barnes maze

The Barnes maze is used to evaluate spatial learning and memory. As previously reported (Wang et al., 2020), mice were trained to locate the escape hole on the Barnes maze over four consecutive days (with three trials/day, each lasting 3 min, and a 15-min interval between each trial). The number of incorrect hole investigations (termed “error”) and the exploration time during each trial were recorded. The platform surface was cleaned with 75% ethanol before each trial to eliminate any odor cues.

During all behavioral testing and scoring processes, experimenters were blinded to the grouping of the animals. Specifically, the animals’ housing cages and testing apparatuses were labeled with unique codes that did not contain any group information. Behavioral videos were scored by the experimenters without access to group information. To avoid carry-over effects, the testing order of each group was randomized. Additionally, appropriate rest intervals were provided between different behavioral tests to minimize the cumulative effects of stress and fatigue from previous tests.

### 2.4 EdU incorporation

To assess cell proliferation and differentiation in the mouse brain after chemotherapy, two intraperitoneal injections of EdU were administered at an interval of 8 h after the end of chemotherapy (Zhang et al., 2021). In adulthood, the mice were euthanized via intraperitoneal injection of pentobarbital sodium (>100 mg/kg), and hippocampal tissues were stained with immunofluorescence to detect EdU^+^ cells and EdU^+^ DCX^+^ cells in the dentate gyrus of the hippocampus.

### 2.5 Immunofluorescence staining

The obtained brains were fixed, immersed in sugar, embedded, and sectioned into 20 μm slices. The sections containing the dentate gyrus were subjected to EdU staining following the instructions of the EdU kit (Cell-Light Apollo Stain Kit, RiboBio, C10310-2). Then, these sections were blocked with 5% BSA and incubated with the anti-DCX antibody (Rabbit anti-DCX, 1:500, Cell Signaling Technology, Massachusetts, United States) overnight at 4 °C. After washing, these sections were incubated with the goat anti-rabbit antibody (1:200, Jackson ImmunoResearch, United States) for 2 h. These sections were treated with an antifluorescence quenching reagent, and the images were acquired using a Zeiss LSM 800 confocal microscope (Carl Zeiss, Jena, Germany).

### 2.6 Western blotting

The frozen hippocampus was homogenized in lysis buffer containing protease inhibitor cocktails (CW2333S, CWBio). Protein concentrations of the samples were determined using a BCA protein assay kit (CW0014S, CWBio) according to the manufacturer’s instructions. Equal amounts of protein were separated by 10% sodium dodecyl sulfate-polyacrylamide gel electrophoresis (SDS-PAGE) and transferred to PVDF membranes. The membranes were blocked with 5% milk in TBST buffer for 1 h and incubated with primary antibodies (rabbit anti-synaptophysin, 1:1,000, 11785-1, Proteintech; rabbit anti-PSD 95, 1:1,000, 3450S, Cell Signaling Technology; rabbit anti-β-tubulin, 1:5,000, Proteintech) overnight at 4 °C. After three washes, the membranes were incubated with the secondary antibody (Goat anti-rabbit IgG, 1:5,000, ab216773, Abcam) at room temperature for 2 h. Finally, visualization of the proteins was performed using the Odyssey CLx imaging system (LI-COR). Relative protein expression levels were normalized by the ratio of target proteins (SYN, PSD95) to β-tubulin.

### 2.7 Microbial 16S rRNA gene sequencing and analysis

Fresh fecal samples were collected at the end of the chemotherapy cycle and in adulthood, and stored at −80 °C for processing. Bacterial 16S ribosomal RNA (rRNA) gene sequencing was used to detect the fecal microbiota composition of the fecal samples. The primer pairs 338-F (5′-ACTACTGGGAGGCAGCAG-3′) and 806R (5′-GGACTACHVGGGTWTCAAT-3′) were used. The PCR amplification products were sequenced on an Illumina MiSeq platform by Majorbio Bio-pharm Technology Co., Ltd. (Shanghai, China). Fecal microbial diversity and abundance across the different samples were analyzed to assess their community structure.

### 2.8 Statistical analysis

Data analysis was performed using SPSS software (version 25.0). The Shapiro–Wilk test was used to assess the normal distribution of the data. If the experimental data followed a normal distribution, they were expressed as “mean ± standard deviation.” Two-way ANOVA was used to analyze the Barnes maze data. One-way ANOVA was used to analyze the data from other behavioral tests across the three groups. Bonferroni’s multiple comparisons test was performed to compare the selected groups when ANOVA showed significance. 16S rRNA sequencing data were analyzed using the Majorbio Cloud platform.^1^ A *t*-test was used to compare the differences between two groups. The statistical significance was set at a *p*-value of <0.05.

## 3 Results

### 3.1 Early-life chemotherapy-induced brain development impairment in the mice

**Figure 1:**
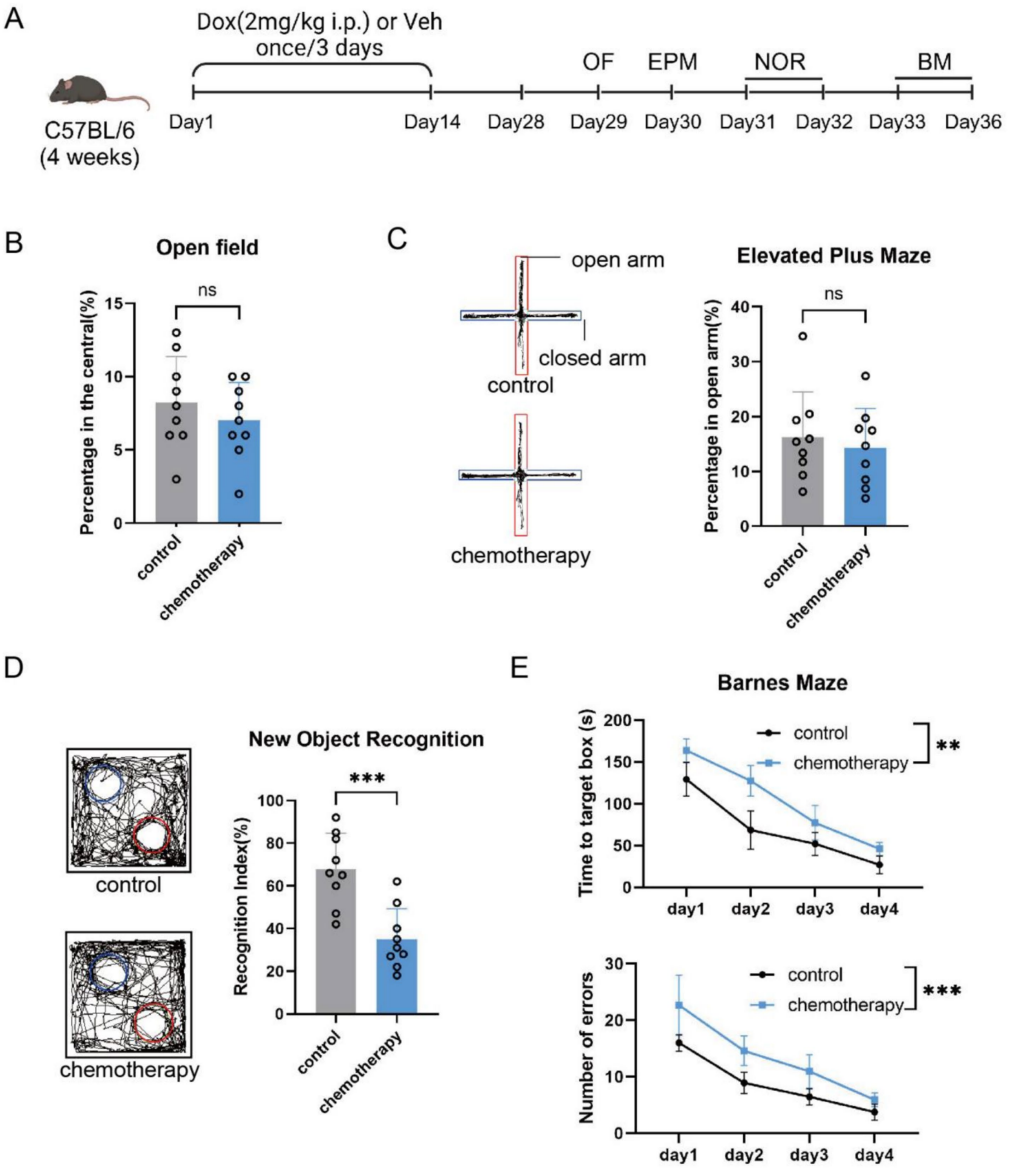
Early-life chemotherapy resulted in cognitive dysfunction in adult mice. **(A)** Schematic showing doxorubicin administration (DOX, 2 mg/kg i.p., once every 3 days for 2 weeks) and behavior tests. **(B)** Time spent in the central area during the open field test (*n* = 9, mean ± SD, *t*-test, *p* = 0.3828). **(C)** Percentage of time spent in the open arms in the elevated plus maze (*n* = 9, mean ± SD, *t*-test, *p* = 0.5949). **(D)** Preference index for a novel object in the novel object recognition task (*n* = 9, mean ± SD, *t*-test, *p* < 0.001). **(E)**. Errors and the time to find the target box in the Barnes maze test (*n* = 9, mean ± SD, two-way repeated measures ANOVA test, ^**^p < 0.01 and ^***^p < 0.001).

To investigate the effects of early-life chemotherapy on adult brain function, 4-week-old mice were injected intraperitoneally with doxorubicin once every 3 days for 2 weeks, and behavioral tests were conducted in adulthood (Figure 1A). Compared to the control group, the chemotherapy-treated mice showed no significant differences in movement and anxiety-like behaviors (*p* > 0.05, Figures 1B,C). However, in the novel object recognition test, the recognition index of the chemotherapy group was significantly lower than that of the control group [*n* = 9, mean (standard deviation), 0.68 (0.17) vs. 0.35 (0.14), *p* = 0.0004, 95% CI (−48.49 to 17.29)] (Figure 1D). In the Barnes maze test, the chemotherapy group required significantly more time to find the target box and made more errors than the control group [*n* = 9, time: *F*(3, 64) = 4.939, *p* = 0.0038, treatment: *F*(1, 8) = 48.24, *p* = 0.0001] (Figure 1E). These results showed that early-life chemotherapy resulted in brain dysfunction in adulthood.

### 3.2 Early-life chemotherapy-induced fecal dysbiosis in mice at the end of chemotherapy and in adulthood

**Figure 2:**
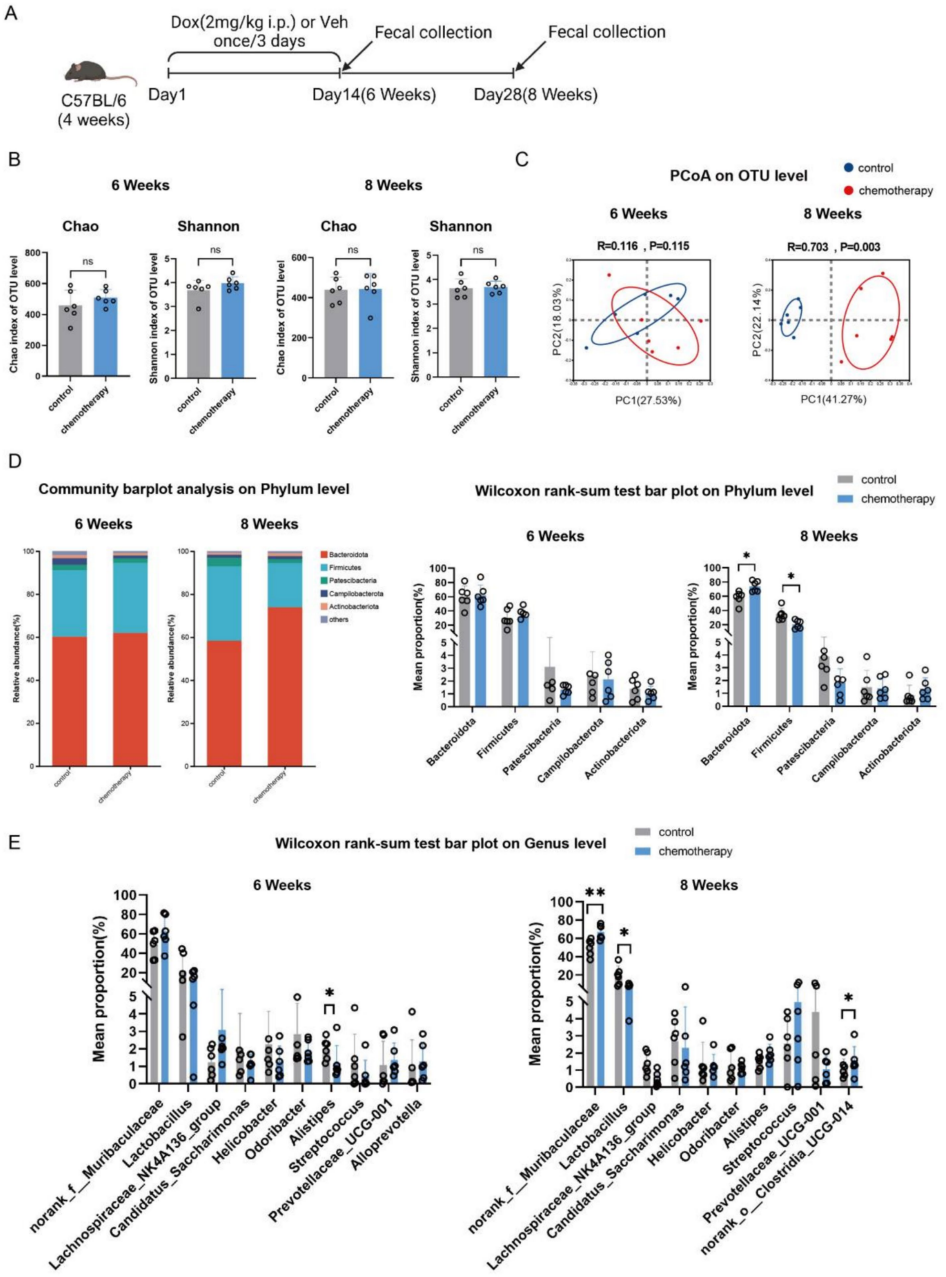
Early-life chemotherapy-induced fecal dysbiosis in mice. **(A)** Schematic showing doxorubicin administration (DOX, 2 mg/kg i.p., once every 3 days for 2 weeks) and fecal collection. **(B)** Microbial community α-diversity (measured by Chao and Shannon indices) (*n* = 6, *p* > 0.05). **(C)** Principal coordinates analysis (PCoA) (*n* = 6; 6 weeks: *p* = 0.115, *R* = 0.116; 8 weeks: *p* = 0.003, *R* = 0.703). **(D)** Community barplot analysis (left panel) and relative abundance (right panel) at the phylum level (*n* = 6, ^*^p < 0.05). **(E)** Relative abundance at the genus level (*n* = 6, ^*^p < 0.05 and ^**^p < 0.01).

Corresponding to the cognitive dysfunction observed in adult mice that underwent early-life chemotherapy, 16S ribosomal RNA (rRNA) gene sequencing showed that at the end of chemotherapy (6 weeks of age), there was no statistical difference between the chemotherapy group and the control group in α-diversity (*p* > 0.05, Figures 2A,B), β-diversity (*n* = 6, *p* = 0.115, *R* = 0.116) (Figure 2C), or species composition and proportion at the phylum level (Figure 2D). At the genus level, the relative abundance of *Alistipes* in the chemotherapy group was significantly decreased (*n* = 6, *p* < 0.05) (Figure 2E). In contrast, in adulthood (8 weeks of age), the chemotherapy group showed statistical differences in β-diversity (*n* = 6, *p* = 0.003, *R* = 0.703) (Figure 2C), the proportion of *Bacteroidetes and Firmicutes* phyla (*Bacteroidetes*, *p* < 0.05; *Firmicutes*, *p* < 0.05) (Figure 2D), and the relative abundance of several genera, including *norank_f_muribaculaceae, Lactobacillus,* and *norank_o_clostridia_UCG-014*, compared to the control group (*norank_f_muribaculaceae*, *p* < 0.01; *Lactobacillus*, *p* < 0.05; and *norank_o_clostridia_UCG-014*, *p* < 0.05) (Figure 2E). These results showed that early-life chemotherapy induced fecal dysbiosis in mice.

### 3.3 Nurturing beneficial fecal microbiota with probiotics improved early-life chemotherapy-induced cognitive impairment

**Figure 3:**
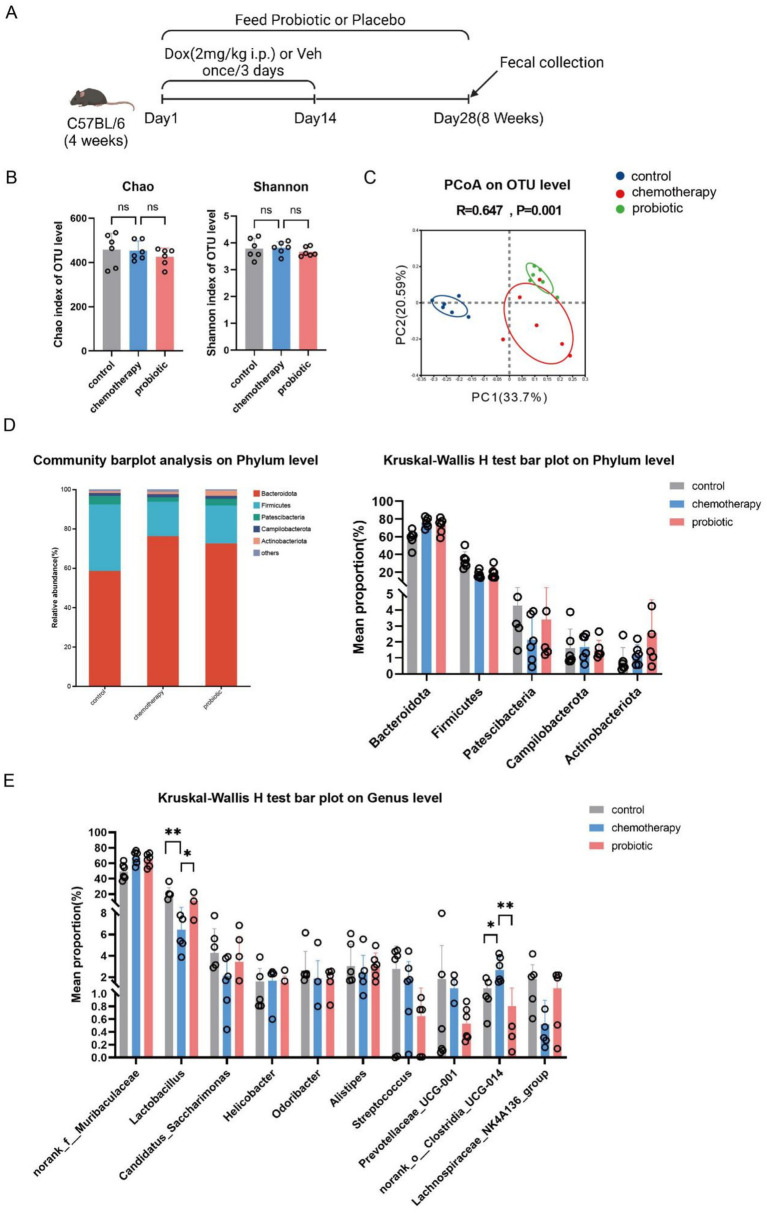
Probiotic supplementation improved early-life chemotherapy-induced fecal dysbiosis. **(A)** Schematic showing doxorubicin administration (DOX, 2 mg/kg i.p., once every 3 days for 2 weeks), probiotic supplementation, and fecal collection. **(B)** Microbial community α-diversity (measured by Chao and Shannon indices) (*n* = 6, *p* > 0.05). **(C)** Principal coordinates analysis (PCoA) (*n* = 6; *p* = 0.001, *R* = 0.647 for three-group comparison; control vs. chemotherapy: *p* = 0.003, *R* = 0.689; chemotherapy vs. probiotic: *p* = 0.001, *R* = 0.993). **(D)** Community barplot analysis (left panel) and relative abundance (right panel) at the phylum level (*n* = 6). **(E)** Relative abundance at the genus level (*n* = 6, ^*^p < 0.05 and ^**^p < 0.01).

**Figure 4:**
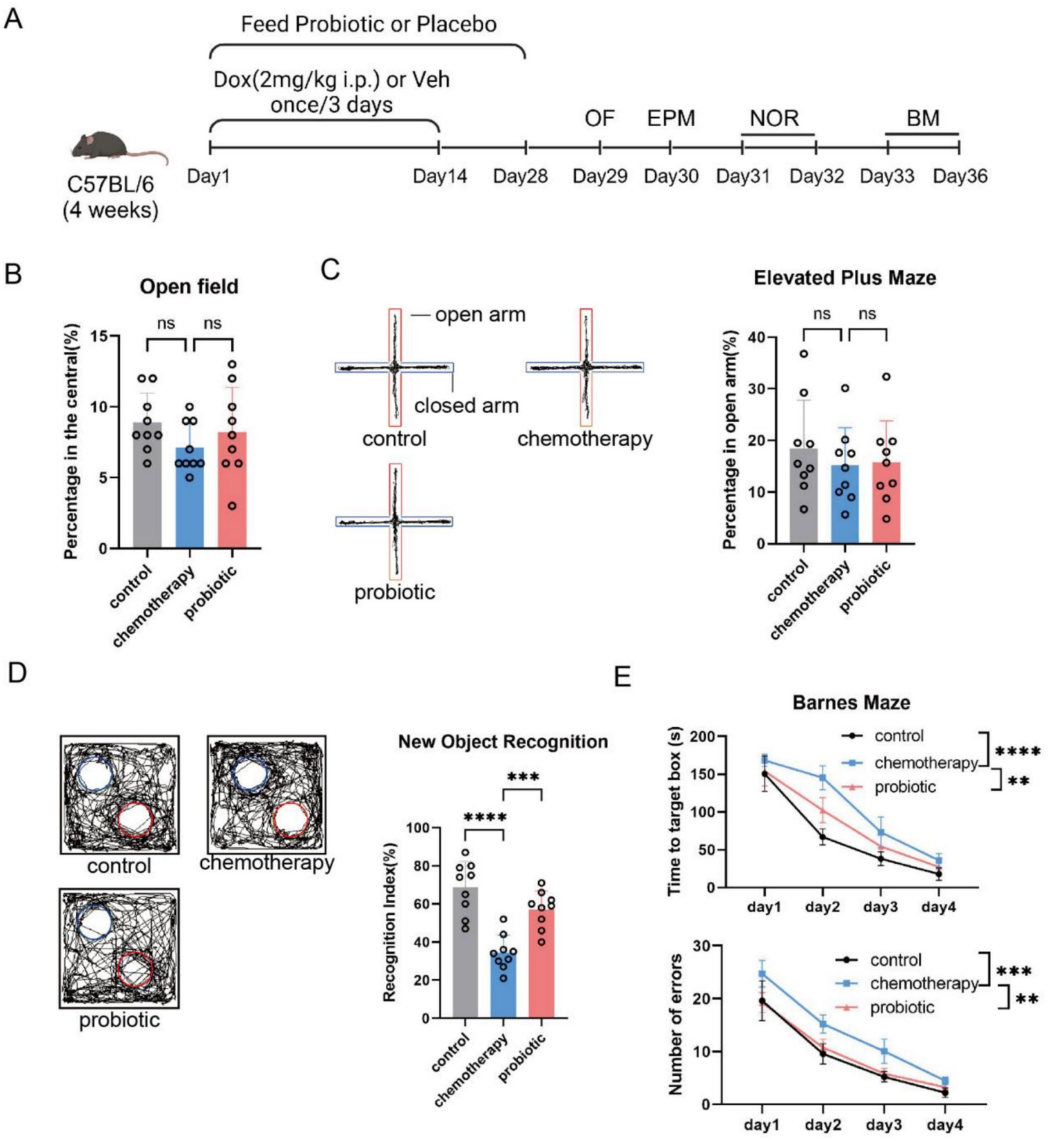
Probiotic supplementation improved early-life chemotherapy-induced cognitive impairment. **(A)** Schematic showing doxorubicin administration (DOX, 2 mg/kg i.p., once every 3 days for 2 weeks), probiotic supplementation, and behavioral tests. **(B)** Time spent in the central area during the open field test (*n* = 9, mean ± SD, AVONA, ns: no significance). **(C)** Percentage of time spent in the open arms in the elevated plus maze (*n* = 9, mean ± SD, AVONA, ns: no significance). **(D)** Preference index for a novel object in the novel object recognition task (*n* = 9, mean ± SD, AVONA, ^***^p < 0.001 and ^****^p < 0.0001). **(E)** Errors and the time to find the target box in the Barnes maze test (*n* = 9, mean ± SD, two-way repeated measures ANOVA test, ^*^p < 0.05, ^**^p < 0.01, ^***^p < 0.001, and ^****^p < 0.0001).

Probiotics are widely used to promote beneficial fecal microbiota. In this study, we administered probiotics to improve early-life chemotherapy-induced fecal dysbiosis and assessed their effects on cognitive impairment (Figure 3A). In adulthood (8 weeks of age), the probiotic group showed statistical differences in β-diversity (*n* = 6, *p* = 0.001, *R* = 0.647; control vs. chemotherapy: *p* = 0.003, *R* = 0.689; chemotherapy vs. probiotic: *p* = 0.001, *R* = 0.993) (Figure 3C), but there were no significant differences in α-diversity among the different groups of mice (*n* = 6, *p* < 0.05. And the relative abundance of *Lactobacillus* and *norank_o_clostridia_UCG-014* (*n* = 6, chemotherapy vs. probiotic: *Lactobacillus*, *p* < 0.05; *norank_o_clostridia_UCG-014*, *p* < 0.01) (Figures 3D,E), compared to the chemotherapy group. We conducted behavioral tests according to the procedure, and found that,there were no significant differences among the three groups of mice in activity and anxiety-like behavior (Figures 4A-C). Corresponding to the improvement in gut dysbiosis, the recognition index of the probiotic group in the novel object recognition test was significantly higher than that of the chemotherapy group [mean (standard deviation), 0.34 (0.09) vs. 0.57 (0.10), *p* = 0006] (Figure 4D). In the Barnes maze test, the probiotic group required significantly less time to find the target box and made fewer errors than the chemotherapy group [time: *F*(3, 64) = 4.597, *p* = 0.0056, treatment: *F*(3, 64) = 5.271, *p* = 0.0026] (Figure 4E). These results showed that probiotic supplementation improved early-life chemotherapy-induced fecal dysbiosis and cognitive impairment.

### 3.4 Probiotic supplementation improved early-life chemotherapy-induced neurogenesis impairment in mice

**Figure 5:**
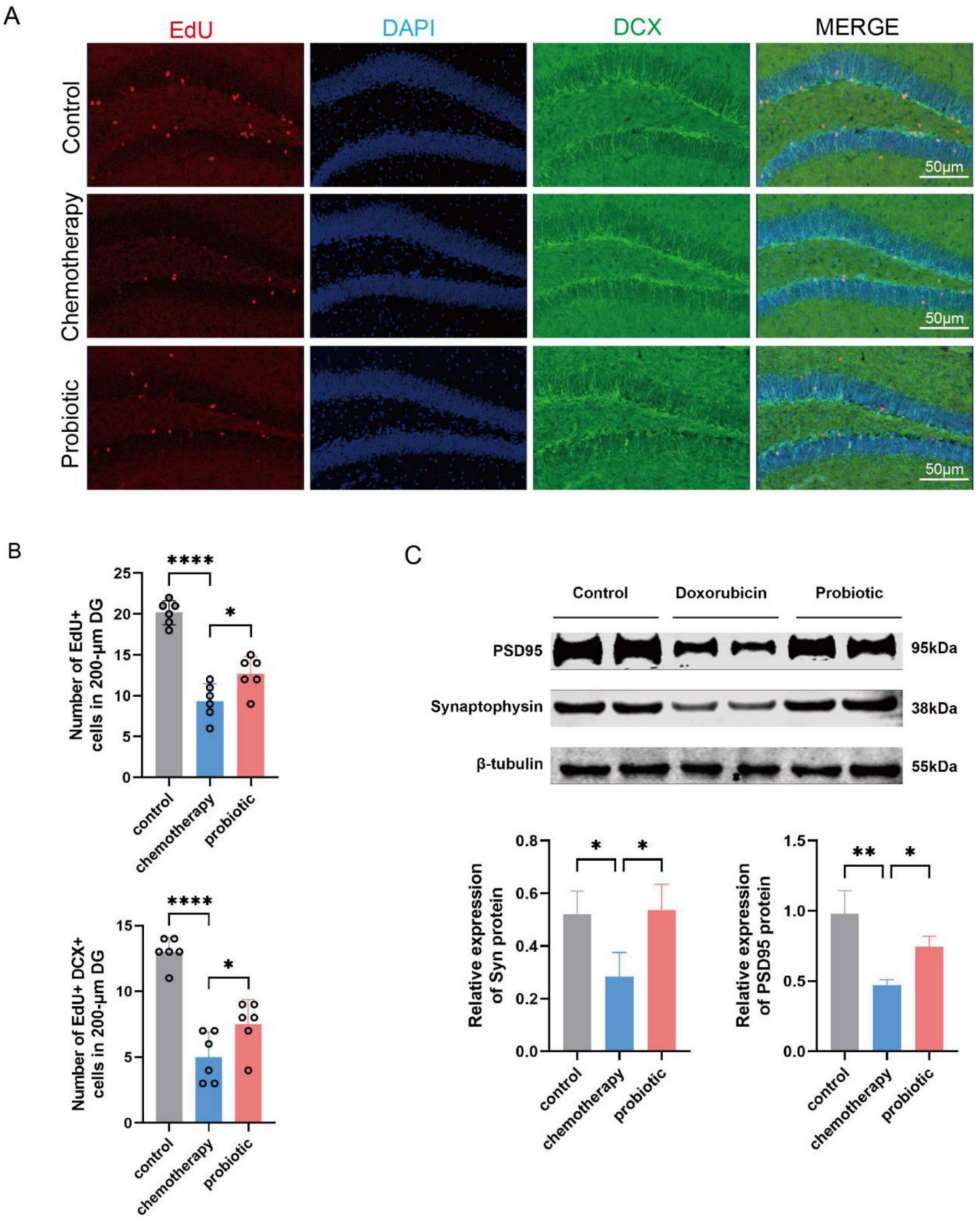
Probiotic supplementation improved chemotherapy-induced reductions in hippocampal neurogenesis and synaptic protein expression. **(A)** Representative images of EdU^+^ (red) and DCX^+^ (green) cells in the dentate gyrus. Bar = 50 μm. **(B)** The number of EdU^+^ cells and EdU^+^ DCX^+^ cells (*n* = 6, mean ± SD, AVONA, ^*^p < 0.05 and ^****^p < 0.0001). **(C)** Western blotting analysis and corresponding quantification of hippocampal proteins (*n* = 4, mean ± SD, AVONA, ^*^p < 0.05 and ^**^p < 0.01).

Neurogenesis is an important characteristic of the developing brain (Terreros-Roncal et al., 2021). To investigate the potential protective mechanisms of probiotics, hippocampal neurogenesis was detected in the control, chemotherapy, and probiotic groups using EdU labeling and doublecortin (DCX) staining. Compared to the control group, the chemotherapy group showed fewer EdU^+^ cells and EdU^+^ DCX^+^ cells in the dentate gyrus [*n* = 6, EdU^+^ cells: mean (standard deviation), 20.17 (1.47) vs. 9.33 (2.16), *p* < 0.0001; EdU^+^ DCX^+^ cells:13.00 (1.10) vs. 5.00 (1.90), *p* < 0.0001] and lower levels of synaptic proteins (Synaptophysin, PSD95) (Syn: *p* < 0.05; PSD95: *p* < 0.01). These changes were found to be significantly improved in the probiotic group [*n* = 6, EdU^+^ cells: mean (standard deviation), 9.33 (2.16) vs. 12.67 (2.16), *p* < 0.05; EdU^+^ DCX^+^ cells: 5.00 (1.90) vs. 7.50 (1.87), *p* < 0.05; Syn: *p* < 0.05; PSD95: *p* < 0.05]. These results showed that probiotic supplementation improved early-life chemotherapy-induced cognitive impairment by modulating neurogenesis (see Figure 5).

## 4 Discussion

Brain dysfunction is a common post-chemotherapy sequela in acute lymphoblastic leukemia (ALL) survivors (Fellah et al., 2019; Krull et al., 2016; Krull et al., 2018). It has a significant impact on the learning and working abilities of ALL survivors (van der Plas et al., 2021b; Cheung et al., 2016; van der Plas et al., 2018). To date, non-pharmacological interventions—such as psychological intervention, behavioral training, and cognitive training—have been used to alleviate cognitive impairments in survivors (Treanor et al., 2016; Fleming et al., 2023). However, poor compliance and the low effectiveness of these measures have led to clinic challenges in treating brain dysfunction in ALL survivors. In this study, we established a clinical ALL chemotherapy mouse model by intraperitoneally injecting doxorubicin during early life and found that probiotic supplementation during and after chemotherapy prevented chemotherapy-induced cognitive function and fecal dysbiosis in adulthood. This is consistent with our previous study involving adult patients with breast cancer (Juan et al., 2022). We found that probiotic supplementation during chemotherapy could alleviate chemotherapy-induced cognitive impairment without causing side effects and with good patient compliance. These data suggest that probiotic supplementation is a practical preventive method for chemotherapy-induced brain dysfunction in ALL children.

The developing brain is easily influenced by environmental factors (Cohen Kadosh et al., 2021). In this study, we found that early-life chemotherapy induced brain development impairments in the mice. Corresponding to these brain impairments, early-life chemotherapy also induced obvious fecal dysbiosis, showing changes in β-diversity and relative abundance of *norank_f_muribaculaceae*, *norank_o_clostridia_UCG-014,* and *Lactobacillus* genera. However, early chemotherapy and probiotic supplementation had no significant effect on the α-diversity of the gut microbiota in any group of mice. At the end of chemotherapy (6 weeks), we found no significant differences in the Chao index or Shannon index among the groups (*p* > 0.05). Similarly, in adulthood (8 weeks), there were no significant differences in the Chao index or Shannon index among the mouse groups (*p* > 0.05). The Chao index is commonly used to estimate species richness that has not been observed, while the Shannon index is used to measure species diversity. These results suggest that chemotherapy had no significant impact on the α-diversity of the mice at the end of chemotherapy or in adulthood. Probiotic supplementation during and after chemotherapy limited early-life chemotherapy-induced fecal dysbiosis, including changes in the β-diversity and relative abundance of *norank_o_clostridia_UCG-014* and *Lactobacillus*, and alleviated early-life chemotherapy-induced hippocampal neurogenesis and brain dysfunction. These data support that enhancement of hippocampal neurogenesis is an important mechanism underlying the effects of probiotics. Accumulated evidence has shown that the gut microbiota can modulate the brain via blood metabolites, the vagus nerve ascending pathway, and the immune system (Wang et al., 2023; Zhang et al., 2022; Bonaz et al., 2018; Loh et al., 2024). Our previous study showed that plasma metabolites, including p-Mentha-1,8-dien-7-ol, play an important role in the protective effects of probiotics against chemotherapy-induced brain injury. Therefore, it is reasonable to infer that plasma metabolites are important mediators in the prevention of early-life chemotherapy-induced brain development impairments by probiotics. However, the true situation needs to be studied further.

Our study has some limitations. First, we did not evaluate the preventive effects of different probiotic formulations. The probiotic mixture tested in this study contained *Bifidobacterium longum*, *Lactobacillus acidophilus*, and *Enterococcus faecalis*. *Bifidobacterium longum* and *Lactobacillus acidophilus* are common probiotic strains. Our findings also revealed that probiotic supplementation increased the relative abundance of *Lactobacillus*. Therefore, our study can be considered a “proof-of-concept” investigation for the prevention of early-life chemotherapy-induced brain development impairments. Second, we only selected doxorubicin for the chemotherapy intervention. Although doxorubicin is a highly effective anthracycline that is widely used in the treatment of various malignant tumors (Mattioli et al., 2023), using a single drug cannot completely replicate the diversity and complexity of treatment regimens in clinical practice. Third, the sample size (*n* = 6) was relatively small for microbiome analysis. The reproducibility of results could be improved with a larger sample size. Fourth, we did not assess the safety of the probiotic used in children undergoing chemotherapy. Probiotics are commonly used in children with diarrhea, and no toxicity has been reported. Theoretically, they should also be safe for children undergoing chemotherapy.

In conclusion, our study found that probiotic intervention can affect hippocampal neurogenesis by modulating fecal microbial composition, thereby improving cognitive impairment in adulthood caused by early-life chemotherapy. These findings provide new insights for the prevention and treatment of chemotherapy-related cognitive dysfunction in children.

## Data Availability

The original contributions presented in the study are included in the article/supplementary material, further inquiries can be directed to the corresponding authors.

## Footnotes

1
http://cloud.majorbio.com
